# Supplementary material for: Deletion of wfs1 Impairs Oligodendrocyte Precursor Cells Dorsal Distribution and Myelination Through the wfs1-hmgcs1 Axis in Zebrafish
Source: Biology (Basel). 2026 Aug 16;15(16):1402. doi: 10.3390/biology15161402 (PMC13510151; doi:10.3390/biology15161402)
Supplement: Supplementary file 1 [file biology-15-01402-s001.zip › File S1-Transcriptome sequencing data and differentially expressed genes (DEGs) in wfs1-- zebrafish at 5 dpf/2.KEGG/wfs1KOvsWT/ALL/src/dre04060.html]

dre04060


- dre:100135062

- Up regulated genes

100135062\_2(1.52157080813253)

- dre:100135062

- Up regulated genes

100135062\_2(1.52157080813253)

- dre:799279

- Up regulated genes

799279(1.8949973992005)

- dre:559723

- Up regulated genes

559723(3.63719210583924)

- dre:768248

- Up regulated genes

768248(6.07490717007993)

- dre:791704

- Up regulated genes

791704(1.64775824422649)

- dre:100001424

- Up regulated genes

100001424(1.37729023397304)

- dre:100135062

- Up regulated genes

100135062\_2(1.52157080813253)

- dre:368438

- Up regulated genes

368438\_1(2.41070971381478)

- dre:100135755

- Up regulated genes

100135755(1.01231209519882)

- dre:562247

- Up regulated genes

562247(7.32702278824855)

- Down regulated genes

562247\_1(-1.84336770590498)

- dre:58151

- Up regulated genes

58151\_1(4.46314096549938)

- dre:799354

- Up regulated genes

799354(4.08509859637481)

- Down regulated genes

799354\_1(-1.21394100025012)

- dre:100002946

- Down regulated genes

100002946(-1.40391573648291)

- dre:100037357

- Down regulated genes

100037357(-1.39102944946719)

- dre:561050

- Up regulated genes

561050\_1(1.31531368091334)

- dre:100006078

- Up regulated genes

100006078(2.32113452971516)

- Down regulated genes

100006078\_1(-5.72416507735977)

- dre:100333914

- Up regulated genes

100333914(2.08181967903621)

- dre:58133

- Up regulated genes

58133(1.32041761049897)

- dre:30125

- Up regulated genes

30125(2.54560579340716)

- dre:368326

- Up regulated genes

368326\_1(4.43392642006339)

- Down regulated genes

368326(-1.63536295102477)

- dre:368326

- Up regulated genes

368326\_1(4.43392642006339)

- Down regulated genes

368326(-1.63536295102477)

Close
